# Sperm H3K9me3 levels are associated with embryo developmental dynamics and biochemical pregnancy in IVF patients with normozoospermia

**DOI:** 10.1186/s12958-025-01505-w

**Published:** 2025-12-03

**Authors:** Kaylee Holleman, Eva S. van Marion, Cindy Eleveld, Elise A. Ferreira, Maria P. H. Koster, Joop S. E. Laven, Willy M. Baarends, Raymond A. Poot, Esther B. Baart

**Affiliations:** 1 Division of Reproductive Endocrinology and Infertility, Department of Obstetrics and Gynaecology, Erasmus MC, University Medical Center, Rotterdam, The Netherlands; 2 Department of Developmental Biology, Erasmus MC, University Medical Center, Rotterdam, The Netherlands; 3 Department of Cell Biology, Erasmus MC, University Medical Center, Rotterdam, The Netherlands; 4 Present address: National Institute for Health and Environment, Bilthoven, The Netherlands

**Keywords:** Normozoospermia, Spermatozoa, Chromatin, Embryonic development, In vitro fertilization

## Abstract

**Background:**

The posttranslational histone modification H3K9me3 is crucial for constitutive heterochromatin (cHC) and supports genome stability and gene regulation during development. This epigenetic mark persists in human sperm post histone-to-protamine transition and is transmitted to the embryo. Although H3K9me3 variability is linked to abnormal sperm parameters, its role in fertilization and embryo development remains unclear. Given its retention in sperm, aberrant H3K9me3 levels may underlie cases of unexplained male infertility.

**Objective:**

Investigate the variability of H3K9me3 levels in sperm from normozoospermic men and assess its association with early embryo development and IVF outcomes.

**Material and methods:**

H3K9me3 and histone H3 levels were quantified by Western blot in surplus sperm from 99 normozoospermic men undergoing IVF-treatment. Patients were stratified into quartiles based on the H3K9me3/H3 ratio. Pre-implantation embryo development was assessed by time-lapse imaging, focusing on nuclear precursor body (NPB) dynamics and morphokinetics. IVF outcomes were reported as cumulative biochemical and ongoing pregnancy rates per ovum pick-up and compared across H3K9me3/H3 quartiles.

**Results:**

H3K9me3/H3 ratios exhibited substantial inter-individual variability among normozoospermic patients. Embryos from the third H3K9me3/H3 ratio quartile (Q3) demonstrated the highest proportion of zygotes with NPB clustering and faster, more consistent development through the first two cleavage divisions compared to other quartiles. A significant non-linear association was found between H3K9me3/H3 ratio and cumulative biochemical pregnancy rates: couples in the lowest quartile (Q1) had significantly reduced odds of biochemical pregnancy compared to Q3 (adjusted OR [95% CI]: 0.30 [0.09–0.97], *p* = 0.045). No significant association was found for ongoing pregnancy rates.

**Discussion and conclusions:**

This study reveals that sperm H3K9me3 levels vary among normozoospermic men and correlate with early embryo development and biochemical pregnancy rates following IVF. However, no significant association was found with ongoing pregnancy, suggesting that additional mechanisms may determine long-term pregnancy viability. The non-linear relationship between H3K9me3/H3 ratio and embryo development suggests an optimal range for this epigenetic mark. These findings highlight the potential influence of paternal epigenetic variation, undetectable by standard semen analysis, on embryo quality and IVF outcomes. Further studies in larger cohorts are warranted to confirm these findings and clarify underlying mechanisms.

**Supplementary Information:**

The online version contains supplementary material available at 10.1186/s12958-025-01505-w.

## Background

During mammalian fertilization, two highly specialized gametes fuse to initiate embryo development. It was once believed that sperm only contributed the haploid male genome to embryo formation, but mounting evidence shows that the sperm epigenome, consisting of chromatin, DNA methylation and non-coding RNAs, is transmitted as well [1]. The oocyte and sperm both have pre-existing and completely different epigenomes, and all layers of epigenetic regulation need to undergo extensive remodelling to ensure proper development of the embryo, including a switch from oocyte control of the transcriptome to the embryo, exit from totipotency, and the emergence of the inner cell mass (ICM) and the extraembryonic lineages.

Epigenetic regulation works by making DNA accessible or inaccessible to proteins such as transcription factors and the transcriptional machinery. To achieve this, DNA is folded with histone proteins to form chromatin. Based on the level of compaction, this chromatin is characterized as euchromatin (accessible) or heterochromatin (inaccessible). Chromatin structure is mostly regulated through histone post translational modifications (PTMs) and the protein machineries that add, remove, or recognize these marks are referred to as writers, erasers, and readers. The constitutive heterochromatin (cHC) forms mainly around the centromeric DNA repeat sequences (satellite DNA), and this type of chromatin is crucial for maintaining genomic integrity and accurate chromosome segregation [2]. The so-called H3K9/HP1 pathway underlies formation and maintenance of cHC, where a central event is tri-methylation of histone H3 at lysine 9 (H3K9me3) [3]. The PTM H3K9me3 is then “read” by heterochromatin protein 1 (HP1) isoforms, resulting in chromatin compartmentalization via phase separation [4]. H3K9me3-mediated compaction of chromatin is a key factor in regulation of cell-fate decisions throughout embryo development [5]. In addition, experimental disruption of the H3K9me3 signature leads to genome instability and chromosome missegregation [6].

Interestingly, after the completion of male meiosis and during spermiogenesis, the histones enfolding the DNA are largely replaced by protamines. This process, generally known as the histone-to-protamine transition, is essential for proper chromatin condensation, and reduces the volume of the sperm nucleus [7, 8], facilitates its mobility and protects the paternal genomic material on the way to and even after fertilization in the zygote [9]. After fertilization, protamines are removed from the paternal chromatin and replaced by oocyte derived histones. Still, during mammalian spermatogenesis some histones are maintained in mature spermatozoa. In mouse, only 1% of the DNA remains histone associated in mature spermatozoa [10], and the paternal H3K9me3 signature at the cHC is mostly removed during mouse spermiogenesis [11]. In human mature spermatozoa, 5–15% of the DNA remains histone associated [12]. We previously showed that in contrast to mouse, human mature spermatozoa still exhibit high levels of H3K9me3 on pericentric repeat regions and transmit these nucleosomes to the embryo after fertilization [13]. These paternal marks are bound by HP1 proteins from the oocyte and propagated over the embryonic cleavage divisions. In mice, paternal constitutive heterochromatin (cHC) is re-established by the oocyte via an alternative pathway that usually controls facultative heterochromatin [11, 14]. However, in humans, paternal cHC appears to be directly defined by histone post-translational modifications (PTMs) inherited from the sperm, making it a paternally regulated feature [13]. An increasing body of evidence supports the idea that sperm-inherited modified histones play an important role in development [1]. For example, paternally inherited H3K9me3 at specific genomic loci was demonstrated to impact embryo development in a mouse model [15]. Therefore, sperm inherited H3K9me3 is likely to impact on genomic integrity and chromosome segregation in the early embryo.

There is increasing evidence that the chromatin content of sperm from subfertile patients is variable. It has been reported that male subfertility patients have an increased nucleosome/protamine ratio compared to fertile patients, indicating incomplete chromatin reorganization during spermiogenesis [5]. This has been associated with poorer IVF outcomes [16, 17]. Using nanoliquid chromatography-tandem mass spectrometry to identify all histone modifications present in mature sperm, it was observed that the relative abundance of different histone PTMs did not vary in normozoospermic patients. However, modifications (no, mono-, di- or trimethylation) on H3K9 formed the exception [18], with a large inter-patient variability. In addition, when comparing sperm H3K9 methylation levels in men with normal semen parameters to men with abnormal semen parameters, a lower relative abundance of H3K9me3 was observed in men with abnormal semen parameters [19]. This variability in the relative abundance of H3K9me3 among sperm samples is noteworthy and indicates patient-specific H3K9me3 levels in sperm. Causes of this variability are largely unknown, but may be related to a defective histone-to-protamine transition, with simply more residual histone H3 that is trimethylated. Alternatively, it can be the result of specific in- or decreased levels of trimethylation on histone H3 during spermiogenesis. This variation in the levels of H3K9me3 in normozoospermic men may be a contributing factor to unexplained male infertility that currently goes undetected in routine sperm quality tests.

A defective histone-to-protamine transition is more frequently observed in men with abnormal semen parameters [20]. To focus on the potential variability in H3K9me3 levels that is not related to a defective histone-to-protamine transition, we limited our study cohort to normozoospermic men. In addition, we aimed to study the impact of variability in the levels of H3K9me3 in sperm on embryo development and pregnancy outcomes. As sperm-inherited H3K9me3 underlies cHC formation in the human zygote [13], variability in the levels of H3K9me3 in sperm is likely to have an impact on cHC function in the early embryo. By first optimizing a method to extract histones from mature human sperm, we were able to assess variation of histone H3 and H3K9me3 content in surplus processed sperm samples by Western blot and correlate this to resulting embryo morphokinetics and pregnancy outcomes.

## Materials and methods

### Study population

This study utilized a prospective cohort design, enrolling couples undergoing in vitro fertilization (IVF) treatment at the Erasmus MC from November 2017 to June 2022. Inclusion criteria were restricted to couples with a male partner exhibiting normozoospermia as defined by WHO criteria (concentration ≥ 15 × 10⁶/mL, progressive motility ≥ 32%, and ≥ 4% normal sperm morphology [21]). Treatment cycles resulting in less than four oocytes after ovum pick-up were excluded. Couples were included only once.

### Ovarian stimulation and oocyte collection

Routine ovarian stimulation was performed by either a GnRH-agonist or –antagonist co-treatment protocol with recombinant- or highly purified urinary- follicle stimulating hormone (FSH; Menopur, Ferring, Gonal-F, Merck Serono, Bemfola, Gedeon Richter Benelux, or Rekovelle, Ferring) as described previously [22]. Final follicular maturation was triggered by the use of human recombinant chorionic gonadotropin (hCG) (Ovitrelle, Merck Serono or Pregnyl, Organon). Ovum pick up was planned according to standardized criteria. Due to changes in routine IVF-practices, oocytes were isolated from the follicular fluid in SAGE 1-HTF (Cooper Surgical, Trumbull) fertilization medium in the period between November 2017 and December 2019, and in G-IVF (Vitrolife) between December 2019 and June 2022, and cultured until insemination.

### Ejaculated sperm processing, selection and oocyte insemination

Ejaculated semen samples were left to liquefy and subsequently processed by density centrifugation, using a 40–80% PureSperm density gradient (Nidacon International AB) or a 45–90% SpermGrad density gradient (Vitrolife) according to the manufacturer’s instructions. Prior to processing, semen volume, sperm concentration, and motility were assessed to evaluate initial total progressive motility in the semen sample (volume x concentration x motility; VCM). The resulting soft pellet was washed once in Sage-HTF-medium or G-IVF medium and the purified spermatozoa were stored at room temperature until insemination. Insemination was performed by adding part of the sperm suspension to the fertilization dish with oocytes in a final concentration of 300.000 sperm cells/ml. Surplus processed sperm cells were kept at RT until protein extraction.

### Embryo culture, embryo transfer, and pregnancy outcome

The day after fertilization, oocytes were denuded and checked for the presence of pronuclei. Zygotes with two pronuclei, as a sign of normal fertilization, were placed in individual wells of EmbryoSlide culture dishes (Vitrolife) and cultured in a time-lapse incubator at 36.8 ℃, 7% O_2_ and 5–6% CO_2_ (EmbryoScope, Vitrolife). Due to changes in routine IVF-practices, embryos were cultured in SAGE 1-step (Cooper Surgical, Trumbull) culture medium between November 2017 and December 2019, and in G-TL (Vitrolife) between December 2019 and June 2022. Embryo transfer was routinely performed on day 3 after fertilization until April 2019. Afterwards, due to a change in routine laboratory and clinical policy, embryo transfer was performed on day 5 after fertilization. Embryo selection for day 3 or day 5 transfer was performed by morphological assessment as described previously [23, 24]. Embryo cryopreservation of surplus good quality embryos was routinely performed using the slow freezing method as previously described on day 4 after fertilization until August 2018 [25]. Afterwards, due to a change in laboratory policy, blastocyst vitrification was performed on day 5 and 6 after fertilization. Blastocysts were frozen and warmed using the RapidVit and RapidWarm Omni kits (Vitrolife, Göteborg, Sweden), according to the manufacturer's instructions. As IVF treatment outcomes, the fertilization rate was calculated as the proportion of fertilized oocytes per cumulus oocyte complex (COC) retrieved. The oocyte and embryo utilization rates were calculated as the number of embryos utilised (i.e. either transferred or cryopreserved) per number of COCs or two-pronuclear (2PN) zygotes generated in the same cycle, respectively. In our clinic it is standard care to transfer a single embryo, however, women aged 38 years or older, or women undergoing their third or higher treatment cycle, can opt for double embryo transfer. Biochemical pregnancy was investigated 10 days after ovum pick up by a urinary β-hCG test and ongoing pregnancy was confirmed by a fetal heartbeat during an ultrasound at 12 weeks of gestation. As the day of transfer can impact embryo selection and may therefore affect pregnancy outcomes, we used cumulative pregnancy rates per treatment cycle as outcome measure. Cumulative pregnancy includes pregnancy outcomes of embryos from the same IVF cycle. Cycle outcomes were followed up to the first biochemical pregnancy and ongoing pregnancy after fresh and any subsequent frozen-thawed embryo transfers. This follow-up continued until all embryos from that cycle were used, with a maximum duration of 2.5 years after oocyte pick-up.

### Time-lapse imaging analysis of embryo development and nucleor precursor body dynamics

The EmbryoScope time-lapse incubator records images automatically in seven focal planes every 15 min. Manual annotations were performed by two trained members of our team according to published consensus definitions and guidelines [26]. Annotations were recorded for number of pronuclei, time to pronuclear fading (tPNf), as well as the timing of reaching the 2, 3, 4, 5, 6, 7, 8-cell stage (t2, t3, t4, t5, t6, t7 and t8). Interval data were calculated. For example t2-tPNf is the time between nuclear fading and the first cleavage division. Nucleolar precursor bodies (NPBs) are specialized structures found within the nucleus of zygotes and their dynamics have previously been associated with implantation potential [27] and chromosome segregation errors such as chromosome misalignment, aneuploidy, micronuclei and impaired embryo development [28]. We analysed NPBs in the pronuclear phase of the zygote based on previously published criteria with minor modifications [28]. For each zygote we determined tPNf and analysed the dynamics of the distinctly visible NPBs. NPB dynamics were analysed between three hours prior to tPNF up to the last frame before tPNf. Starting from the frame three hours before tPNf, NPBs are statically classified based on the positioning within the pronuclei. The junction between the two pronuclei determines the axis where the NPBs should align. When NPBs are found at the junction of the pronuclei the static classification is ‘clustered’. If one or more of the NPBs are scattered within the pronucleus or not aligned at the PN junction, the NPBs are statically classified as ‘unclustered’. In addition to the NPB clustering dynamics analyses we also included the static classification of the clustering status of the NPBs on the last frame prior to tPNf.

### Histone extraction from surplus human spermatozoa

Histone proteins from mature sperm cells were extracted by incorporating DTT during protein extraction (see below) to reduce disulfide bonds between protamines and facilitate lysis. Directly after insemination, the surplus purified sperm suspension was centrifuged for 7 min at 200 × *g*. Supernatant was removed and the pellet was washed in 1 × PBS to dilute the sample before counting in duplo on a Makler counting chamber for semen concentration calculation and a check for round cell contamination. Samples with round cell contamination were not included. Samples were only included when there were at least 5.6 *10^6^ sperm left after processing and routine IVF insemination, in order to have enough material for technical duplicates of the Western blot. After the concentration was determined, the samples were again centrifuged for 7 min at 200 × *g* and the sperm pellet diluted in RIPA Buffer (R0278, Sigma Aldrich) with 5 µM DTT and 1 × Halt™ Protease Inhibitor Cocktail (10,085,973, Thermo Fisher Scientific) to a concentration of 200 × 10^6^ spermatozoa/ml. Samples were incubated on ice for 30 min. Subsequently, samples were sonicated using a Bioruptor (UCD-200, Diagenode) at power 5 (high) for 15 min with 30 s pulses (30 s pause between pulses) at 4 °C, while samples were immersed in an ice bath. Next, samples were vortexed for 5 min and centrifuged for 20 min at 13.000 × *g* at 4 °C. Finally, the supernatant was pipetted into 1.5 ml protein LoBind Eppendorf tubes and stored at −20 °C for later use. Protein quantification using a BSA protein assay kit could not be performed reliably due to presence of DTT in the lysis buffer.

### Western blot analysis of histone H3 and H3K9me3

Protein extracts were thawed at 37 °C for 5 min and then mixed with 4 × Laemmli sample loading buffer (1,610,747, Bio-Rad) containing 5% 2-mercaptoethanol at a ratio of 3 parts protein extract to 1 part buffer. Denaturation was performed at 95 °C for 5 min. To determine the optimal sample loading volume for western blot, we tested a range of volumes (several microliters per lane) using random sperm protein lysate samples. Band intensities for histone H3 and H3K9me3 were quantified to identify the volume that produced a strong, unsaturated signal within the linear detection range. Based on this analysis, 7 µL per sample was selected for loading in duplicate onto 4–20% precast gradient gels (ID-PA4201-012, Eurogentec). On each gel, a Precision Plus Protein Dual Color Standards marker (1,610,374, Bio-Rad) was included, as well as 1.7 µg purified histone H3 protein (ab198757, Abcam) as a quality control for the blotting efficiency. Proteins were separated using electrophoresis at 100 V for 75 min (Mini PROTEAN Tetra System, Bio-Rad). Proteins were then transferred from the gel onto a 0.2 µm nitrocellulose membrane (1,620,112, Bio-Rad) for 1 h at 100 V (Mini Trans-blot cell, Bio-Rad). Subsequently, the membranes were rinsed in water, dried for 10 min at 37 °C, and rehydrated in TBS for total protein staining (TPS, 926–11,016, Li-Cor). Total protein staining, scanning, and destaining were performed according to the manufacturer's recommendations.

For immunostaining, membranes were incubated in blocking buffer TBST with 5% skim milk (70,166, Sigma Aldrich) for 1 h at room temperature. To detect H3K9me3 at the N-terminus of histone H3, a previously validated antibody was used (rabbit-anti-Tri-Methyl-Histone H3 (Lys9) (D4W1U), Cell Signalling, 1:1000) [13]. To avoid potential competition of antibody binding, an antibody against the C-terminus of human histone H3 was selected for the detection of core histone H3 (Histone H3 (96C10) Mouse mAb Cell Signaling, #3638, 1:1000). These primary antibodies were diluted in blocking buffer and incubated overnight at 4 °C. After washing with TBST, membranes were incubated with secondary antibodies IRDye 680RD donkey anti-mouse (926–68,072, Li-Cor) and IRDye 800CW goat anti-Rabbit (926 32,211, Li-Cor) for 1.5 h at room temperature. Membranes were imaged on the Odyssey Clx scanner at 700 and 800 nm using the standardized intensity and image-quality settings (sFig. 1). Scans of TPS and immunostainings were analyzed using the Li-Cor Image Studio Lite program. For TPS scans, no background subtraction was applied. For all samples, TPS showed a band around 67 kDa that was highly variable in intensity, likely caused by residual human serum albumin (66.5 kDa) present in the culture medium and sperm processing gradients (sFig1A). To mitigate variations, only the area below ~ 50 kDa was measured. Intensity measurements of H3 and H3K9me3 bands (15 kDa) were performed using equal areas and background subtractions.

### Histone H3 levels, H3K9me3/H3 ratio calculation and data validation

Sperm protein lysates from each patient were analyzed in technical duplicates by running two separate gels, resulting in a total of 28 gels and blots. To ensure reproducibility of electrophoresis and blotting, the intensity of the histone H3 reference protein (H3ref) bands from all blots was measured. The average band intensity and standard deviation (SD) of H3ref across all blots were calculated. Any blot with an H3ref band intensity outside the range of the overall mean ± 2 × SD was excluded from further analysis. For normalization, the relative expression of histone H3 was calculated by dividing the H3 band intensity by the total protein stain intensity in the same lane, then multiplying by 100 to express the result as a percentage. The ratio of H3K9me3 to H3 was determined by dividing the H3K9me3 band intensity by the corresponding H3 band intensity within the same lane.

These calculations were performed separately for each technical replicate. The variation between duplicates (duplo) was then assessed by calculating the difference between replicates for each sample. The average variation and SD across all samples were determined. Patient samples with duplicate variation outside the range of the mean ± 2 × SD were excluded from the study to ensure data reliability. Per sample, the average of normalized histone H3 content and the H3K9me3/H3 ratio from the technical duplicates was taken for further analysis on embryo morphokinetics and IVF outcome.

Within the study period, the IVF-laboratory changed the routine procedure for sperm preparations from a 80/40% sperm processing gradient to a 90/45% sperm processing gradient. Histone H3 levels were significantly decreased when sperm was processed with a 90/45% gradient (Mann Whitney U, *p*-value = 0.0002) (sFig. 2A). However, no significant differences were observed for the H3K9me3/H3 ratios between the two sperm processing methods (sFig. 2B), and there was also no effect on fertilization rate (sFig. 2 C). Therefore, we did not perform corrections based on the sperm processing gradients in further analysis.

### Statistical analysis

Baseline characteristics and treatment outcomes of all included cycles with a result for relative H3 and H3K9me3/H3 ratio were tested for normal distribution. Because continuous data was not normally distributed, a Mann–Whitney U test was performed to compare data between the group with- and without a biochemical pregnancy. Estimates are reported as medians and interquartile range (IQR). Categorical data were analysed with the Chi-square test/Fisher exact test. Spearman ranked correlation coefficients were calculated to assess the correlation between two continuous variables. To analyse the effect of H3K9me3 levels in sperm on embryo morhokinetcis and pregnancy outcome, patients were categorized into four equal sized quartile groups (Q1-Q4), with Q1 representing the lowest 25% and Q4 the highest 25% of H3K9me3/H3 ratio values. Embryos from the same couple are likely to show similar developmental characteristics and thus show clustering [29]. Also, the developmental time points of one embryo cannot be considered as independent measurements. Therefore, to analyze the association between the H3K9me3/H3 ratio in sperm and pre-implantation embryo development, time-lapse data was analyzed using a linear mixed model approach to correct for this clustering and to take the repeated measurements into account. Model 1 included the sperm H3K9me3/H3 ratio and a fixed effect adjustment for patient-ID to account for measures of multiple embryos from one couple [29]. Model 2 extended model 1 by additionally including adjustments for type of culture medium as this has previously been shown to impact on embryo developmental morphokinetics [30]. The association between cumulative biochemical (positive hCG), and cumulative ongoing pregnancy (12 weeks gestation) and the H3K9me3/H3 ratio was analysed using a logistic regression analysis with addition of the confounder female age. All statistical analyses were performed in the statistical package for the social sciences (SPSS), version 28.0.1.0. Two-sided *p*-values < 0.05 were considered significant.

## Results

### Patient characteristics

**Fig. 1:**
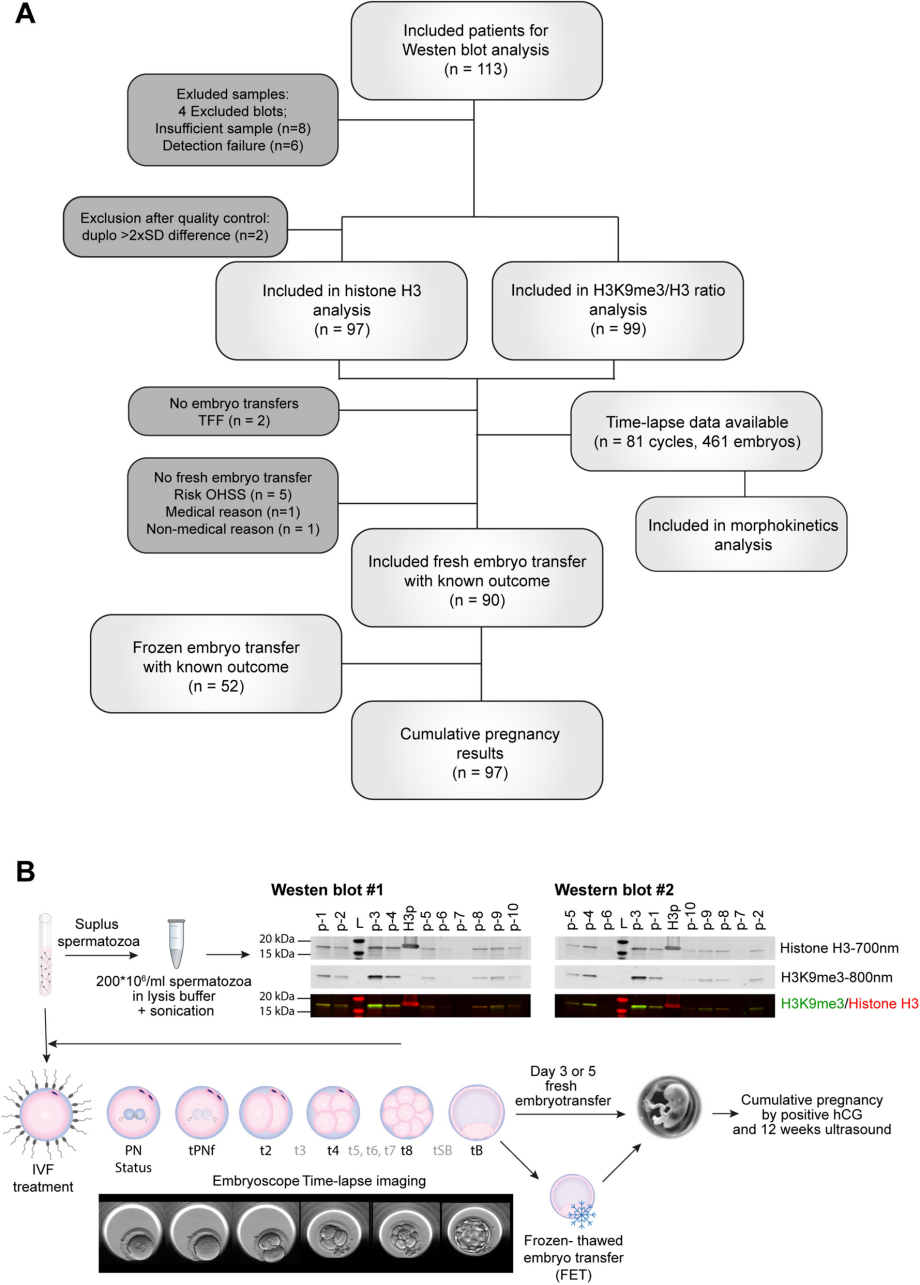
Flowchart patient inclusion and exclusion per analysis and schematic overview of the prospective observational study. **A** Abbreviations: SD, Standard deviation; TFF, Total fertilization failure; OHSS, ovarian hyper-stimulation syndrome. **B** Routine IVF treatment was performed. Resulting 2PN zygotes were transferred to the Embryoscope time-lapse incubator after pronuclear examination. Embryos were cultured up to either day 3 or day 5 according to routine IVF protocols. Morphokinetic parameters were annotated up to tB for comparison of the H3K9me3/H3 ratios. Surplus sperm samples were lysed and used for Western blot analysis in technical duplicates. Histone H3 and H3K9me3 were detected with Near-infrared (NIR) fluorescence at 700 nm and 800 nm, respectively, to allow quantification. Abbreviations: (L) protein ladder, (H3p) recombinant histone H3 protein

A total of 113 IVF-couples were included with surplus sperm samples available for protein extraction (Fig. 1A). A schematic overview of the experimental set up is presented (Fig. 1B). Samples from 14 cycles were excluded in the final analysis due to insufficient sperm cells available or measurements of either H3 of H3K9me3 that were below the threshold of the background substraction. Thus, in 99 samples, lysis and Western blot analysis was successful so that H3 levels and the H3K9me3/H3 ratio could be determined. Baseline characteristics of these included IVF-cycles are shown (sTable 1). In 90 cycles a fresh embryo transfer was performed. In the other 9 cycles, fresh transfer was not possible due to a risk of ovarian hyperstimulation syndrome (OHSS, *n* = 5), no embryos available due to total fertilization failure (*n* = 2), and medical or personal reasons (*n* = 2). Cumulative pregnancy results, meaning the combined pregnancy outcomes from the fresh and all subsequent frozen-thawed embryo transfers from the same cycle, were available for 97 cycles up to ongoing pregnancy. The embryos (*n* = 461) from 81 cycles underwent culture in the EmbryoScope time-lapse incubator and were available for morphokinetic analysis of embryo development.

### Sperm Histone H3 and H3K9me3 levels in relation to male age and IVF outcomes

Western blot analysis of surplus spermatozoa was performed to investigate the impact of H3K9me3 levels on embryo morphokinetics and IVF outcomes. The histone H3 content in sperm samples was determined by normalizing H3 band intensities over total protein staining and ranged between 0.002 and 0.050 arbitrary units (AU) (median 0.013 AU and IQR 0.010—0.020 AU). H3K9me3 content was then expressed as H3K9me3 band intensity over H3 band intensity and ranged between 0.014 and 2.498 AU (median 0.133 AU and IQR 0.075—0.260 AU) in our study group of IVF patients. To analyse the correlation between the histone H3 content, H3K9me3/H3 ratio, male age and VCM (volume x concentration x motility) a Spearman ranked correlation was performed (sFig 3). No significant correlations were found between male age and histone H3 (ρ = 0.078, *p*-value 0.449)(sFig. 3 A) or the H3K9me3/H3 ratio (ρ = −0.112, *p*-value 0.230)(sFig. 3B). Additionally, no significant correlations were observed for VCM and histone H3 (ρ = 0.110, *p*-value 0.283)(sFig 3 C) or H3K9me3 (ρ = 0.173, *p*-value 0.087)(sFig. 3D). A significant positive correlation was observed between histone H3 level and the H3K9me3/H3 ratio (ρ = 0.205, *p*-value 0.044). Furthermore, patient characteristics of clinical features and treatment outcomes were compared across the H3K9me3/H3 ratio quartiles (Table 1). No significant differences were found in patient characteristics or clinical features. However, in the treatment outcomes a trend is observed for the embryo usage rate per oocytes retrieved (EUR/COC) and per fertilized oocyte (EUR/2PN) being higher for Q3 (transfer and cryopreserved) in the IVF-treatment cycle (Table 1). From the 99 cycles with successful western blot results, resulting embryos of 81 cycles were cultured in a time-lapse incubator. This resulted in time-lapse data of 461 embryos, of which 81 embryos were transferred, 228 cryopreserved and 152 discarded. These data were used in the subsequent analyses of nucleolar precursor body clustering and morphokinetics.

**Table 1:** Patient characteristics and IVF-treatment outcome in H3K9me3/H3 quartile groups

	H3K9me3/H3 ratio Q1 (lowest-0.075) (*n* = 25)	H3K9me3/H3 ratio Q2 (0.075–0.133) (*n* = 24)	H3K9me3/H3 ratio Q3 (0.133–0.260) (*n* = 25)	H3K9me3/H3 ratio Q4 (0.260-highest) (*n* = 25)	*p*-value
Male age	38.33 [34.32–40.49]	37.79 [31.62–40.72]	35.17 [32.66–40.87]	36.17 [32.96–38.79]	0.563
Female age	36.58 [31.67–39.54]	36.17 [32.06–38.56]	36.75 [32.62–39.42]	35.08 [30.63–38.58]	0.843
Main couple diagnosis					
Unexplained infertility	15	7	10	8	0.327
Fallopian tube factor	3	6	5	6	
Irregular menstrual cycle	1	4	2	0	
PCOS	0	1	0	3	
Endometriosis	5	4	7	4	
Uterine factor	0	1	0	1	
Other	1	1	1	3	
VCM	105.01 [70.35–162.06]	99.83 [62.14–136.86]	165.03 [119.74–257.51]	120.12 [74.10–229.93]	0.050
Histone H3	0.013 [0.010–0.020]	0.012 [0.010–0.017]	0.014 [0.010–0.019]	0.018 [0.013–0.025]	0.133
Number of oocytes	8.00 [5.00–11.00]	6.5 [5.25–11.00]	8.00 [6.00–10.00]	8.00 [5.00–13.50]	0.753
Fertilization rate	64.71 [40.00–80.00]	72.12 [50.00–83.33]	66.67 [51.67–80.00]	60.00 [42.81–78.46]	0.476
Oocyte utilization rate	33.33 [20.00–50.00]	42.59 [34.09–65.00]	46.67 [34.85–63.33]	40.00 [25.00–56.44]	0.095
Embryo utilization rate	54.55 [50.00–75.00]	76.39 [55.54–97.50]	83.33 [52.78–100.00]	73.21 [60.63–95.00]	0.097
Time lapse culture	17 (79 embryos)	22 (134 embryos)	22 (122 embryos)	20 (126 embryos)	
*Culture medium*					
*SAGE 1-step* *G-TL*	*1 (3 embryos)* *16 (76 embryos)*	*8 (40 embryos)* *14 (94 embryos)*	*2 (7 embryos)* *20 (115 embryos)*	*9 (63 embryos)* *11 (63 embryos)*	**< 0.001**
Cumulative biochemical pregnancy (within the same cycle) (*n* = 97)					0.101
Yes	9 (36%)	14 (58%)	16 (64%)	17 (68%)	
no	16 (64%)	10 (42%)	9 (36%)	8 (32%)	
Cumulative ongoing pregnancy (within the same cycle) (*n* = 97)					0.611
Yes	8 (32%)	12 (50%)	9 (36%)	11 (44%)	
No	17 (68%)	12 (50%)	16(64%)	14 (56%)	

Data are reported as median [interquartile range (IQR)]

*Abbreviations*: *PCOS* Poly cystic ovary syndrome, *VCM* Volume*concentration*motility, *COC* cumulus oocyte complex, *2PN* two pronuclei containing zygote

*p*-value of < 0.05 was considered significant

### Nucleolar precursor body clustering analysis during the pronuclear stage

**Fig. 2:**
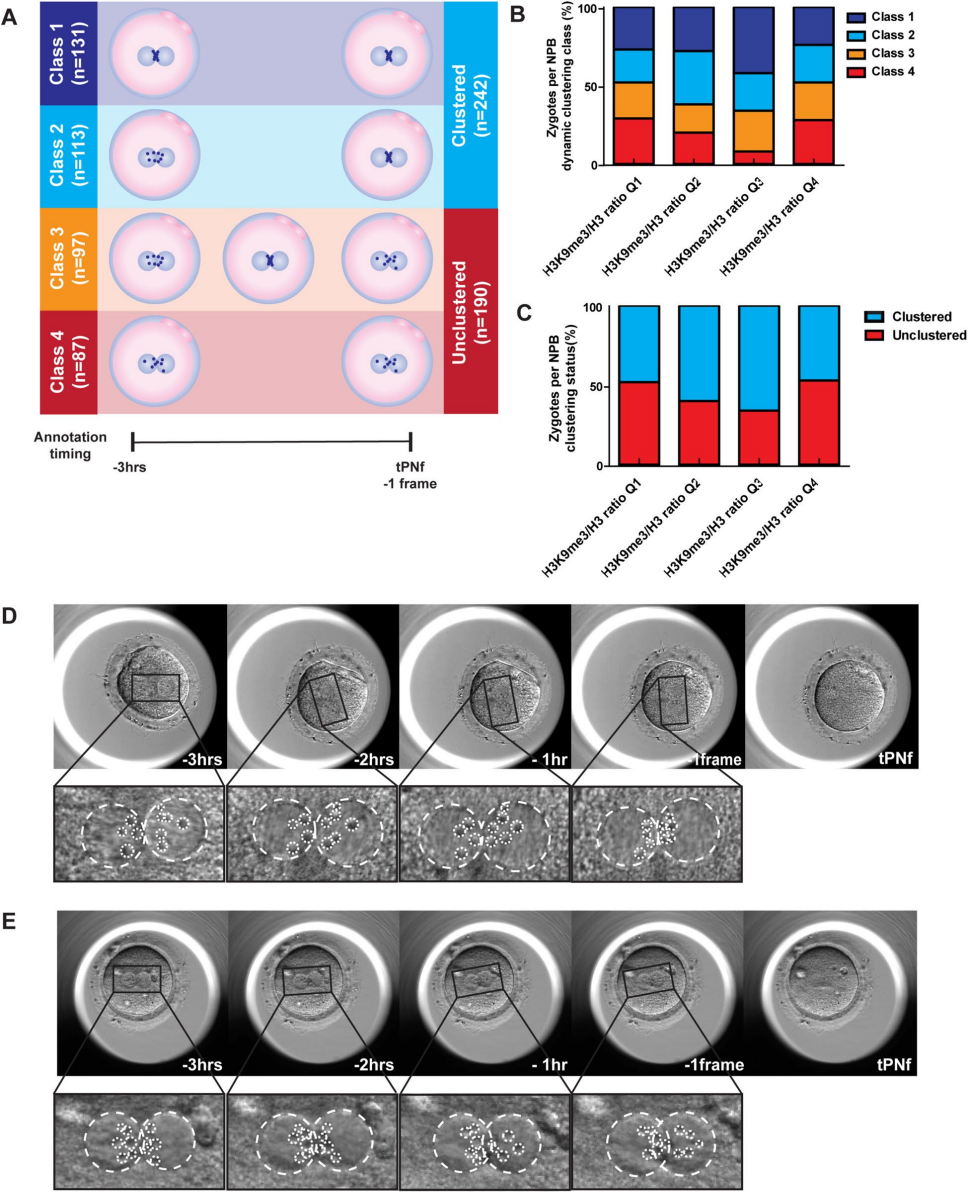
Nucleolar precursor body (NPB) clustering analysis prior to the time of pronuclear fading (tPNf). **A** Schematic overview of nucleolar precursor body analysis and classification. To determine NPB dynamics, zygotes were observed during the 3 h before pronuclear fading (tPNf) and classified as follows: in class 1 zygotes, the NPBs are already aligned 3 h before tPNf and remain at that position. In class 2, the NPBs were scattered throughout the PN but align towards the PN junction within 3 h. In class 3, NPBs start scattered, then and align, but scatter and lose their alignment right before tPNf. In class 4, the NPBs are scattered throughout the PN and stay in an unclustered state before tPNf. Based on a static view of the last frame before tPNf, class 1 and 2 zygotes are categorized as clustered and class 3 and 4 zygotes as unclustered. **B**,**C** Nucleolar precursor body clustering in zygotes in relation to the H3K9me3/H3 ratio observed in the sperm sample divided in quartiles (sTable 2 and 3): quartile 1 (*n* = 17, 73 zygotes), quartile 2 (*n* = 22, 126 zygotes), quartile 3 (*n* = 22, 113 zygotes) and quartile 4 (*n* = 20, 116 zygotes). **B** NPB clustering classified by dynamic assessment. **C** NPB clustering classified by static assessment. **D**, **E** Representative time-lapse images of the 3 h leading to tPNf showing a class 2 zygote with unclustered NPBs that subsequently align; and (**E**) a class 3 zygote where the NPBs become unaligned shortly before tPNf

Previous work from our group showed that the cHC at the pronuclear stage is mainly located surrounding the nucleolar precursor bodies (NPBs) in human zygotes [13]. We therefore aimed to explore if there is a relation with clustering of the NPBs during the pronuclear phase and the sperm H3K9me3/H3 ratio. To explore this, we used the time-lapse data set and a previously published classification system [28]. In addition to the three previously published categories, we observed an intermediate NPB clustering class in our data that was not described previously. Thus, we classified NPB clustering dynamics into four categories (Fig. 2A) [28]. In class 1, NPBs are already localized at the pronuclear axis and remain at that position during the 3 h before tPNf (sVideo 1). In class 2, the NPBs align at the pronuclear axis within 3 h before tPNf (sVideo 2), whereas in class 3 (newly defined) the NPBs initially align, but then become unclustered (sVideo 3). In class 4, the NPBs remain in their original unclustered position or even move further away from the pronuclear axis (sVideo 4). Next to NPBs dynamic classification, an additional static evaluation of NPB alignment was made to determine if the NPBs were clustered or unclustered on the last frame before tPNf (Fig. 2A). We were able to classify NPB dynamics in a time span between 3 h prior to tPNf and tPNf in 428 zygotes (Fig. 2A,B, sTable 3). In some zygotes we were unable to monitor the NPB movement due to unavailable data when frames were out of focus or < 3 h of frames before tPNf were available for analysis (*n* = 33). In 432 zygotes the last frame before pronuclear fading showed clearly visible pronuclei and enabled classification of the static clustering status just before tPNf (Fig. 2A, C, sTable 4). In some zygotes we were unable to analyse the static NPB alignment as the last frame prior to tPNf was unavailable when the frame remained out of focus (*n* = 29).

To study whether the H3K9me3/H3 ratio influences the NPB clustering dynamics, we used the H3K9me3/H3 ratio quartile groups. Previous work showed that zygotes that show clustering of the NPBs and maintain their clustered status up to the moment of tPNf are less likely to have misalignment or aneuploidy during the first cell division [28]. NPB clustering dynamics showed that sperm assigned to Q3 gave rise to the highest percentage zygotes that already achieved NPB clustering 3 h before tPNf (class 1; 42% vs. 27, 28, 24%). This analysis shows that there is a significant difference among the quartiles for the NPB clustering dynamics classes (χ^2^; *p*-value: 0.001) (sTable 3), but also for the static NPB clustering assessment based on the last frame before tPNf (χ^2^; *p*-value: 0.011) (sTable 4). Overall, zygotes from H3K9me3/H3 Q3 showed the highest percentage of zygotes that have NPBs with the ability to move towards the pronuclear axis (class 1–3). Interestingly, H3K9me3/H3 Q1 and 4 show reduced proportions of zygotes with optimal NPB clustering dynamics (NPB movement class 1 and 2). Importantly, Q1 and Q4 also show the highest proportions of unclustered NPBs prior to tPNf (52% and 53%, respectively). This suggests an optimal sperm H3K9me3/H3 ratio in relation to the clustering of NPBs in the pronuclear phase of the resulting zygote.

### Association between H3K9me3/H3 ratio and embryo developmental morphokinetics

Using the time-lapse annotation data, we also investigated the association between the sperm H3K9me3/H3 ratio and histone H3 content with embryo developmental morphokinetics. A linear mixed model analysis was performed to evaluate the relation between the timing of embryo development and quartile categories of the sperm H3K9me3/H3 ratio. Initial data exploration using scatter plots showed the distribution of embryos according to developmental timings. A group of embryos could be identified that showed direct unequal cleavage (DUC), meaning they divided to three cells within 5 h after reaching the 2-cell stage [31, 32]. However, the distribution of these DUC embryos did not differ between the quartiles and we therefore did not correct for this (sTable 5). The box-plots of embryo developmental time-points show that among the H3K9me3/H3 quartiles, Q3 exhibited the narrowest interquartile range (IQR) in the embryo developmental timings, especially for the first two embryonic cell cycles (ECC) (sFig. 4). As Q3 also showed the most optimal NPB clustering and embryo usage rate (EUR), this was taken as the reference group. We analysed the data using linear mixed modelling and used two model

Linear mixed model 1, adjusting only for clustering of embryos from one couple, revealed significant associations between the sperm H3K9me3/H3 quartiles and developmental morphokinetics of the resulting embryos on tPNf, t2, t3, t4 and t4-tPNf. There was an overall trend that development of embryos in Q1, Q2 and Q4 proceeded slower over the first two cell divisions than the reference quartile (Q3) as evidenced by a higher β in hours (Table 2). Compared to Q3, Q4 exhibited a significant developmental delay in the timing of pronuclear fading (tPNf) (β = 1.597 h, 95% CI 0.001, 3.192, *p* = 0.050) as well as a delay in reaching the 2-cell stage (t2)(β = 1.859 h, 95% CI 0.150, 3.569, *p* = 0.033) and 3-cell stage (t3)(β = 2.158 h, 95% CI 0.171, 4.145, *p* = 0.034)(Table 2). Q2 was significantly slower than Q3 at the 2-cell stage (t2)(β = 1.758 h, 95% CI 0.067, 3.448, *p* = 0.042) and 3-cell stage (t3)(β = 2.158 h, 95% CI 0.171, 4.145, *p* = 0.034)(Table 2). Development to the the 4-cell stage (t4) was significantly delayed in embryos from Q1, (β = 2.873 h, 95% CI 0.343, 5.402, *p* = 0.027), Q2 (β = 2.788 h, 95% CI 0.502, 5.074, *p* = 0.018) and Q4 ((β = 2.608 h, 95% CI 0.299, 4.917, *p* = 0.028)). For later developmental stages up to reaching the 8-cell stage, no significant differences among quartiles were found.

**Table 2:** Linear mixed model analysis, Model 1: H3K9me3/H3 ratio in association with time-lapse morphokinetics of resulting IVF-embryos

	H3K9me3/H3 ratio Q1 (lowest-0.075) Beta (95%CI), hours	*p*-value	H3K9me3/H3 ratio Q2 (0.075–0.133) Beta (95%CI), hours	*p*-value	H3K9me3/H3 ratio Q3 (0.133–0.260)	H3K9me3/H3 ratio Q4 (0.260-highest) Beta (95%CI), hours	*p*-value
tPNf	1.547 [−0.158, 3.253]	0.075	1.264 [−0.293, 2.822]	0.110	ref	1.597 [0.001, 3.192]	**0.050**
t2	1.609 [−0.230, 3.449]	0.085	1.758 [0.067, 3.448]	**0.042**	ref	1.859 [0.150, 3.569]	**0.033**
t2-tPNf	0.132 [−0.552, 0.816]	0.702	0.393 [−0.230, 1.016]	0.211	ref	0.391 [−0.243, 1.024]	0.222
t3	1.810 [−0.362, 3.983]	0.101	1.965 [0.002, 3.929]	**0.050**	ref	2.158 [0.171, 4.145]	**0.034**
t3-tPNf	0.442 [−1.180, 2.063]	0.588	0.874 [−0.568, 2.316]	0.230	ref	0.554 [−0.918, 2.027]	0.454
t4	2.873 [0.343, 5.402]	**0.027**	2.788 [0.502, 5.074]	**0.018**	ref	2.608 [0.299, 4.917]	**0.028**
t4-tPNf	1.537 [−0.072, 3.145]	0.061	1.348 [−0.070, 2.766]	0.062	ref	0.854 [−0.591, 2.299]	0.242
t5	0.633 [−2.321, 3.587]	0.670	1.943 [−0.717, 4.602]	0.149	ref	1.073 [−1.601, 3.747]	0.425
t5-tPNf	−0.646 [−3.126, 1.834]	0.605	0.994 [−1.195, 3.182]	0.367	ref	−0.356 [−2.588, 1.875]	0.751
t8	0.797 [−3.299, 4.893]	0.699	1.490 [−2.168, 5.149]	0.417	ref	0.211 [−3.505, 3.927]	0.910
t8-tPNf	0.083 [−3.458, 3.625]	0.963	0.484 [−2.618, 3.587]	0.755	ref	−1.359 [−4.551, 1.134]	0.397
ECC 2 (t4-t2)	1.502 [−0.059, 3.063]	0.059	1.123 [−0.268, 2.513]	0.112	ref	0.706 [−0.696, 2.108]	0.318
ECC 3 (t8-t4)	−1.409 [−4.268, 1.450]	0.328	−0.862 [−3.345, 1.619]	0.487	ref	−1.467 [−4.022, 1.087]	0.254

Model outputs are reported as β coefficients, representing the estimated difference (in hours) for embryos to reach a given developmental time point or interval, relative to the reference quartile, according to the H3K9me3/H3 ratio. Values in parentheses indicate the 95% confidence interval (CI), reflecting the range in which the true effect is expected to lie with 95% probability.)]. Model 1 only takes clustering of embryos from each couple into account

*Abbreviations*: *tPNf* fading of the pronuclei; *t2* time of cleavage of the embryo to the 2-cell stage, *t2-tPNf* time between tPNf and t2, *t3* time of cleavage of the embryo to the 3-cell stage, *t3-tPNf* time between tPNf and t3, *t4* time of cleavage of the embryo to the 4-cell stage, *t4-tPNf* time between tPNf and t4, *t5* time of cleavage of the embryo to the 5-cell stage, *t5-tPNf* time between tPNf and t5, *t8* time of cleavage of the embryo to the 8-cell stage, *t8-tPNf* time between tPNf and t8, *ECC 2* timing of embryonic cell cycle 2 (t4-t2; timing between the 2-cell stage and the 4-cell stage), *ECC 3* timing of embryonic cell cycle 3 (t8-t4; timing between the 4-cell stage and the 8-cell stage)

A *p*-value of < 0.05 was considered significant

After adjustment for type of culture medium in model 2 we still observed the trend that embryos from Q1, Q2 and Q4 are slower in completing the first two cell divisions compared to Q3 as evidenced by a higher β in hours (Table 3). However, only the delay in reaching the 4-cell stage (t4) remained significant for Q1 (β = 2.888 h, 95% CI 0.343, 5.432, *p* = 0.027) and Q2 (β = 2.579 h, 95% CI 0.210, 4.947, *p* = 0.033). In addition the interval between t4-tPNf as well as the interval of the second embryonic cell cycle (ECC2; t4-t2) was longer for Q2 (t4-tPNf: β = 1.633 h, 95% CI 0.208, 3.058, *p* = 0.025; t4-t2: β = 1.463 h, 95% CI −0.083, 2.844, *p* = 0.038)(Table 3). Again, no differences were observed for cleavage timings from the 4-cell to the 8-cell stage in model 2. Overall, the sperm H3K9me3/H3 ratio seems to be associated with embryo morphokinetics up to the 4-cell stage, but not for later developmental time points.

**Table 3:** Linear mixed model analysis, Model 2: H3K9me3/H3 ratio in association with time-lapse morphokinetics of resulting IVF-embryos

	H3K9me3/H3 ratio Q1 (lowest-0.075) Beta (95%CI), hours	*p*-value	H3K9me3/H3 ratio Q2 (0.075–0.133) Beta (95%CI), hours	*p*-value	H3K9me3/H3 ratio Q3 (0.133–0.260)	H3K9me3/H3 ratio Q4 (0.260-highest) Beta (95%CI), hours	*p*-value
tPNf	1.600 [−0.031, 3.230]	0.054	0.756 [−0.774, 2.285]	0.328	ref	0.839 [−0.777, 2.455]	0.304
t2	1.666 [−0.097, 3.429]	0.064	1.234 [−0.426, 2.894]	0.142	ref	1.064 [−0.672, 2.800]	0.225
t2-tPNf	0.135 [−0.552, 0.823]	0.695	0.359 [−0.283, 1.001]	0.268	ref	0.333 [−0.344, 1.010]	0.329
t3	1.818 [−0.373, 4.009]	0.102	1.882 [−0.156, 3.919]	0.070	ref	2.027 [−0.102, 4.157]	0.062
t3-tPNf	0.387 [−1.147, 1.920]	0.616	1.332 [−0.064, 2.728]	0.061	ref	1.248 [−0.231, 2.728]	0.097
t4	2.888 [0.343, 5.432]	**0.027**	2.579 [0.210, 4.947]	**0.033**	ref	2.294 [−0.172, 4.760]	0.068
t4-tPNf	1.506 [−0.068, 3.079]	0.060	1.633 [0.208, 3.0.58]	**0.025**	ref	1.292 [−0.214, 2.798]	0.091
t5	0.576 [−2.517, 3.668]	0.711	2.186 [−0.630, 5.002]	0.126	ref	1.445 [−1.670, 4.561]	0.358
t5-tPNf	−0.656 [−3.024, 1.711]	0.582	1.640 [−0.501, 3.781]	0.131	ref	0.698 [−1.586, 2.983]	0.543
t8	0.820 [−3.306, 4.945]	0.692	1.700 [−2.143, 4.945]	0.379	ref	0.487 [−3.554, 4.528]	0.810
t8-tPNf	−0.191 [−3.286, 3.668]	0.913	1.203, [−1.964, 4.369]	0.448	ref	−0.265 [−3.651, 3.121]	0.876
ECC 2 (t4-t2)	1.459 [−0.053, 2.971]	0.058	1.463 [−0.083, 2.844]	**0.038**	ref	1.242 [−0.208, 2.692]	0.092
ECC 3 (t8-t4)	−1.362 [−4.222, 1.497]	0.345	−0.516 [−3.106, 2.073]	0.690	ref	−0.968 [−3.730, 1.793]	0.485

Model outputs are reported as β coefficients, representing the estimated difference (in hours) for embryos to reach a given developmental time point or interval, relative to the reference quartile, according to the H3K9me3/H3 ratio. Values in parentheses indicate the 95% confidence interval (CI), reflecting the range in which the true effect is expected to lie with 95% probability. Model 2 takes clustering of embryos from each couple into account as well as culture media brand

*Abbreviations*: *tPNf* fading of the pronuclei, *t2* time of cleavage of the embryo to the 2-cell stage, *t2-tPNf* time between tPNf and t2, *t3* time of cleavage of the embryo to the 3-cell stage, *t3-tPNf* time between tPNf and t3, *t4* time of cleavage of the embryo to the 4-cell stage, *t4-tPNf* time between tPNf and t4, *t5* time of cleavage of the embryo to the 5-cell stage, *t5-tPNf* time between tPNf and t5, *t8* time of cleavage of the embryo to the 8-cell stage, *t8-tPNf* time between tPNf and t8, *ECC 2* timing of embryonic cell cycle 2 (t4-t2; timing between the 2-cell stage and the 4-cell stage), *ECC 3* timing of embryonic cell cycle 3 (t8-t4; timing between the 4-cell stage and the 8-cell stage)

A *p*-value of < 0.05 was considered significant

### The impact of histone H3 levels and the H3K9me3/H3 ratio on pregnancy outcome

We next examined the association of the levels of histone H3 and the H3K9me3/H3 ratio on cumulative pregnancy outcomes. Baseline characteristics of the IVF patients and the cycle included in the study are presented for those that did or did not result in biochemical pregnancy after either the fresh or frozen-thawed embryo transfer (Table 4). We observed a significant difference in female age (Mann–Whitney U test; *p*-value: 0.026), the number of oocytes (Mann–whitney U test; *p*-value: 0.001) and embryo usage rate (*p*-value: 0.018) between the cumulative biochemical pregnant and non-pregnant group. As female age is a well-known confounder for pregnancy outcome, we corrected for that in the logistic regression analysis. A binary logistic regression was performed with the sperm H3K9me3/H3 ratio included as a continuous predictor of cumulative implantation with female age as a covariate. In the adjusted model, the H3K9me3/H3 ratio was not significantly associated with cumulative biochemical pregnancy (OR = 1.52, 95% CI 0.50–4.66, *p* = 0.462)(sTable 6). In addition, the same analysis was performed with histone H3 as main determinant for biochemical pregnancy with female age as covariate and again no association was found (OR = 1.16, 95% CI 0.79–1.72, *p* = 0.454) (sTable 7). We repeated this analysis for cumulative ongoing pregnancy, and this also did not show an association for either the H3K9me3/H3 ratio or Histone H3 (sTable 8, 9 and 10).

**Table 4:** Baseline characteristics of patients IVF-cycles resulting in cumulative biochemical pregnancy or no biochemical pregnancy

	No biochemical pregnancy (*n* = 42)	Cumulative biochemical pregnancy (*n* = 55)	*p*-value
Male age	38.00 [33.96–40.21]	36.25 [32.66–40.70]	0.448
Female age	37.50 [34.71–39.83]	34.67 [31.25–37.89]	**0.026**
Main diagnosis			
Unexplained infertility	16	23	0.157
Fallopian tube factor	5	15	
Irregular menstrual cycle	5	2	
PCOS	1	2	
Endometriosis	12	8	
Uterine factor	0	2	
Other	3	3	
Number of oocytes	6.0 [5.0–8.5]	9.5 [6.0–12.75]	**0.001**
Fertilization rate	62.50 [40.00–81.82]	69.23 [50.00–80.00]	0.200
Embryo Utilization Rate	58.58 [44.44–85.12]	75.0 [66.7–100.0]	**0.018**
Embryo transfer			
SET	37	51	0.495
DET	5	4	
Histone H3	0.014 [0.010–0.020]	0.014 [0.010–0.021]	0.541
H3K9me3/H3 ratio	0.11 [0.06–0.24]	0.16 [0.09–0.34]	0.107
Day of cryopreservation			
Day 4	9	10	0.798
Day 5,6	33	45	

Data are reported as median [interquartile range (IQR)]

*Abbreviations*: *PCOS* Poly cystic ovary syndrome, *SET* Single embryo transfer, *DET* Double embryo transfer

A *p*-value of < 0.05 was considered significant, in bold

To investigate a potential non-linear relationship between H3K9me3/H3 ratio and cumulative biochemical pregnancy, we conducted a multivariable binary logistic regression using quartile 3 (Q3) as the reference group and with female age as covariate. We observed that women in Q1 exhibited significantly lower odds of achieving a biochemical pregnancy after a fresh and any additional frozen embryo transfers compared to those in Q3 (Q1: OR = 0.30, 95% CI 0.09–0.97, *p* = 0.045)(Table 5). Conversely, no significant differences were found for the other quartiles compared to Q3. We observed no significant differences across quartiles for the cumulative ongoing pregnancy rates (sTable 11).

**Table 5:** Logistic regression analysis; H3K9me3/H3 ratio quartiles as main determinant and cumulative biochemical pregnancy as outcome

				95% C.I. for OR	
	Beta	OR	*p*-value	lower	Upper
H3K9me3/H3 ratio quartiles					
Quartile 1 vs. Quartile 3	−1.206	**0.30**	**0.045**	0.09	0.97
Quartile 2 vs. Quartile 3	−0.294	0.75	0.623	0.23	2.40
Quartile 4 vs. Quartile 3	0.102	1.11	0.866	0.34	3.64
Female age	−0.082	0.92	0.092	0.84	1.01

Odds ratios adjusted for female age

Q3 was selected as reference category based on exploratory findings

*Abbreviations*: *OR* odds ratio, *C.I.* confidence interval

A *p*-value of < 0.05 was considered significant, in bold

## Discussion

By optimizing a protocol for extracting histones from mature human sperm cells, we successfully quantified histone H3 and its trimethylated form, H3K9me3, using Western blot analysis in a prospective cohort of IVF couples. While we observed some inter-patient variability in histone H3 levels, the H3K9me3/H3 ratio exhibited substantially greater variability among our cohort of good-prognosis, normozoospermic patients. Notably, the sperm H3K9me3/H3 ratio showed a non-linear association with both zygotic nucleolar precursor body dynamics and embryo morphokinetics up to the 4-cell stage. Embryos derived from sperm samples in the third quartile of the H3K9me3/H3 ratio distribution, demonstrated the highest proportion of zygotes with clustered NPBs indicative for lower risk of aneuploidy. They also showed morphokinetic characteristics associated with higher embryo quality [28, 33]. Additionally, this group exhibited a trend toward a higher proportion of embryos suitable for transfer or cryopreservation, as reflected by an increased embryo utilization rate. In addition, we observed that women whose partners’ sperm samples fell into the third quartile had significantly increased odds of achieving a biochemical pregnancy compared to those in the first quartile. However, no significant association was observed between the H3K9me3/H3 ratio and cumulative ongoing pregnancy rates.

In contrast to our observations, a recent study reported that higher histone H3 content in sperm from normozoospermia patients was associated with reduced fertilization, embryo quality and pregnancy outcome after ICSI [34]. However, no association was found between these outcomes and H3K9me3 levels in the same study. This study comprised a small sample size and used a dot-blot assay for analysis. This does not involve electrophoresis to separate proteins prior to detection, making it impossible to assess possible aspecific binding of the antibodies to other proteins in the sample. Furthermore, the normalization approach used in the Dot-blot analysis was based on total protein levels, which could introduce sample-to-sample variability. In our study, we observed an evident band at ~ 65 kDa after total protein staining, which is most likely caused by residual human serum albumin from the culture medium, prompting us to exclude this region in our histone H3 normalization strategy. Moreover, because histone levels and their post-translational modifications (PTMs) vary greatly between sperm samples, normalizing histone PTMs to their corresponding total histone levels—rather than to total protein—provides a more accurate and reliable measurement.

During spermatogenesis, male germ cells first undergo mitosis and meiosis to generate haploid round spermatids. These spermatids undergo spermiogenesis, when histones bound to the DNA are replaced with protamines and the chromatin becomes highly compacted in the sperm head [35]. Little is known regarding the molecular mechanisms underlying the remodelling process, and what regulates this exchange process. More importantly, it remains largely unknown how histone retention is regulated and how histone post-translational modifications affect these dynamics [7, 36, 37]. This becomes even more important as environmental, toxic and dietary exposures have been shown to alter histone retention profiles in sperm, and influence epigenetically inherited traits [38–40]. In our study, we observed a 25-fold variation in overall sperm-retained histone H3, compared to a much larger, 150-fold variation in levels of H3K9me3. This wide variation may reflect differences or defects in the histone-to-protamine transition during spermiogenesis. Notably, we found little correlation between the amount of retained H3 and the H3K9me3/H3 ratio, indicating that histone retention alone does not explain the observed variability in H3K9me3 levels. These findings suggest that the regulatory mechanisms controlling histone retention and those governing histone post-translational modifications operate through distinct pathways.

Interestingly, H3K9me3 is also involved in DNA damage detection and plays a role in recruiting DNA repair proteins to sites of damage. The presence of H3K9me3 at double strand breaks (DSBs) contributes to the recruitment and activation of the DNA damage response cascade [41, 42]. During spermiogenesis, DSBs are intentionally introduced to relax the DNA and facilitate histone removal and protamine incorporation. This controlled DNA damage induction and subsequent repair must be tightly regulated to preserve genomic integrity. When errors occur during this chromatin reconfiguration, DNA damage may persist, leading to elevated H3K9me3 levels specifically at damage sites without necessarily changing overall H3 levels, thereby altering the H3K9me3 to H3 ratio. In our study, we did not assess DNA damage directly, and further research is needed to clarify the relationship between H3K9me3 and DNA damage in human sperm.

Previous studies have shown that the first cleavage divisions of the human embryo are error-prone [43–46]. To investigate if variation in the sperm H3K9me3/H3 ratio affects this, we first focused on nucleolar precursor body (NPB) clustering in the zygote as a non-invasive read-out associated with aneuploidy [28]. Our analysis revealed significant differences in NPB clustering dynamics across H3K9me3/H3 quartiles. Both low and high sperm H3K9me3 levels appeared suboptimal for proper NPB alignment, suggesting an ideal level of H3K9me3 for optimal 3D heterochromatin organization in the zygote. In model systems, experimental removal of H3K9me3 from pericentromeric heterochromatin resulted in a significant reduction in centromeric localization of protein complexes involved in chromosome segregation regulation, such as the chromosomal passenger complex (CPC), and subsequent chromosome malsegregation [47–49]. While investigating chromosome segregation regulation in human zygotes, we previously observed that maternal chromatin, which is globally enriched in H3K9me3, can recruit higher levels of the chromosomal passenger complex (CPC) under experimental conditions [50]. In contrast, CPC recruitment to paternal chromosomes was reduced and limited to the few H3K9me3-positive constitutive heterochromatin (cHC) regions. This finding highlights the critical role of H3K9me3 in CPC recruitment in the zygote. Since sperm-inherited H3K9me3 drives cHC formation in the zygote, we hypothesize that variability in sperm H3K9me3 levels may affect the fidelity of chromosome segregation for the paternal chromosomes. Further research is needed to clarify the role of sperm inherited constitutive heterochromatin in chromosome segregation at this stage.

We next studied the impact of variation in the sperm H3K9me3/H3 ratio on embryo developmental timings up to the 8-cell stage using linear mixed modelling. The first model only adjusted for sibling embryos showed that embryos in the third quartile proceeded significantly faster to the 4-cell stage than embryos from the other quartiles. A previous study from our group showed that the early cleavage divisions are susceptible to culture media composition [30]. Over the study period in the current paper two types of single step media were used (G-TL and SAGE 1-step) during routine IVF practices. To investigate if this change affected our analysis, we adjusted for the type of culture media used in model 2. Here, the same overall trend was observed as in model 1. Delays as observed by time-lapse imaging have been shown to be predictive for embryo aneuploidy in human embryos [33], and could therefore be related to putative chromosome alignment problems caused by a suboptimal cHC signature on paternal chromosomes as mentioned above. In addition, recent studies on human preimplantation embryos have demonstrated a crucial role for H3K9me3 in stage-specific expression regulation of retrotransposons, a type of constitutively repressed heterochromatic elements [51]. An experimentally induced in- or decrease of H3K9me3 at retrotransposon DNA resulted in defective embryonic genome activation and poor embryo development. For future research, it would be interesting to assess H3K9 methylation of retrotransposons in sperm chromatin to understand its impact on early embryo development and successful progression through embryogenesis.

We observed no correlations between H3K9me3 levels and later-stage embryo developmental kinetics after correction for culture medium type. However, we noted a trend toward higher embryo usage rates in the third quartile group, suggesting better embryo quality. The observed developmental delays occurring before the 8-cell stage and embryonic genome activation may lead to reduced developmental potential to the blastocyst stage. This is in line with established associations between sperm chromatin packaging (measured by histone-to-protamine ratios or DNA accessibility) and blastocyst development [52, 53]. Given these connections, a plausible correlation between sperm chromatin packaging and H3K9 trimethylation levels warrants further investigation.

Our analysis also revealed a significant non-linear association between the sperm H3K9me3/H3 ratio and cumulative biochemical pregnancy rates. Specifically, patients in the lowest ratio quartile demonstrated markedly reduced odds of biochemical pregnancy compared to those in the third quartile. This observation suggests that an intermediate (i.e., third quartile) level of sperm H3K9me3 may represent a more favorable epigenetic state that supports early embryo developmental competence and implantation. In contrast, the absence of a significant association between H3K9me3/H3 ratio and ongoing pregnancy rates implies that this paternal epigenetic impact may be limited to the earliest stages of development. Beyond implantation, outcomes are likely determined by additional maternal and embryonic factors such as maternal physiology, embryonic genetic integrity, uterine receptivity and placental development, which may overshadow the effect of sperm-derived histone modifications. Clinically, these findings suggest that assessing sperm H3K9me3 levels could potentially serve as an early biomarker of early embryo development and implantation potential, though not necessarily of sustained pregnancy success. Nevertheless, given the relative small sample size and wide confidence intervals within quartiles, these results should be interpreted with caution and warrant validation in larger cohorts.

To specifically investigate variation in H3K9me3 levels independent of a defective histone-to-protamine transition, our study cohort was limited to normozoospermic patients. Still, a previous clinical study using mass spectrometry reported greater variation in H3K9me3 relative abundance among men with abnormal semen parameters compared to normozoospermic individuals [19]. It is possible that this increased variation in relative abundance would translate to a wider distribution of H3K9me3/H3 ratios, potentially concentrating more patients in the extreme high or low ratio groups. Consequently, a study population including men with abnormal semen parameters might reveal a clearer association between H3K9me3 level variability and IVF outcomes. However, our Western blot approach required sufficient surplus spermatozoa to generate enough lysate for at least duplicate analysis, restricting patient inclusion. Another limitation was that we measured H3K9me3 levels as the H3K9me3/H3 ratio in lysates derived from bulk sperm samples. This method cannot assess the ratio within the individual spermatozoon that fertilized the oocyte. So, our conclusions on the influence of H3K9me3 on human embryo development and implantation of the embryo assume that patients exhibiting a high H3K9me3/H3 ratio in the bulk sample are also more likely to possess individual spermatozoa with higher ratios. Quantifying histones and their modifications in smaller samples, or ideally single sperm cells, would require more sensitive and non-invasive techniques.

## Conclusions

Despite its low prognostic value for male fertilizing potential, both in vivo and in assisted reproductive techniques, semen analysis remains the cornerstone of the diagnosis of male infertility. This poor predictive value is particularly relevant in couples with unexplained infertility, who experience reproductive difficulties despite showing normal sperm parameters. Here we provide evidence that in this group of patients, certain variations in sperm chromatin parameters may affect early embryo development, but go undetected by sperm quality tests currently in use. The increased use of in vitro fertilization treatment underscores the need to improve our knowledge of factors impacting the paternal epigenome and the downstream effects in the embryo.

## Supplementary Information

Supplementary file 1. Supplementary figure legends 1–4 and supplementary tables 1–11.

Supplementary file 2: Figure S1. Representative images of Western blot with 10 patient samples confirming antibody specificity and protein size.

Supplementary file 3. Figure S2. Variation in histone H3 levels and H3K9me3/H3 ratios between 80/40% and 90/45% sperm processing gradient.

Supplementary file 4. Figure S3. Spearman rank correlation between histone H3 level, H3K9me3/H3 ratio, male age and VCM.

Supplementary file 5. Figure S4. Boxplots of embryo morphokinetic annotations up to t8 (time to 8-cells stage) per H3K9me3/H3 quartile.

Supplementary file 6. Video S1. Representative video of a zygote showing nucleolar precursor body (NPB) clustering dynamics class 1.

Supplementary file 7. Video S2: Representative video of a zygote showing nucleolar precursor body (NPB) clustering dynamics class 2.

Supplementary file 8. Video S3: Representative video of a zygote showing nucleolar precursor body (NPB) clustering dynamics class 3.

Supplementary file 9. Video S4: Representative video of a zygote showing nucleolar precursor body (NPB) clustering dynamics class 4.

## Data Availability

The data underlying this article cannot be shared publicly due to the privacy of individuals that participated in the study. The data will be shared on reasonable request to the corresponding author.

## Abbreviations

2PN: Two Pronuclei
AU: Arbitrary Units
CI: Confidence Interval
COC: Cumulus-Oocyte Complex
CPC: Chromosomal Passenger Complex
cHC: Constitutive Heterochromatin
DNA: Deoxyribonucleic Acid
DSB: Double-Strand Break
DTT: Dithiothreitol
DUC: Direct Unequal Cleavage
EUR: Embryo Usage Rate
FSH: Follicle-Stimulating Hormone
GnRH: Gonadotropin-Releasing Hormone
H3: Histone H3
H3K9me3: Histone H3 Lysine 9 Trimethylation
H3ref: Reference Histone H3
hCG: Human Chorionic Gonadotropin
HP1: Heterochromatin Protein 1
ICM: Inner Cell Mass
ICSI: Intracytoplasmic Sperm Injection
IQR: Interquartile Range
IVF: In Vitro Fertilization
kDa: Kilodalton
MEC: Medical Ethics Committee
NPB: Nucleolar Precursor Body
OHSS: Ovarian Hyperstimulation Syndrome
OR: Odds Ratio
PBS: Phosphate-Buffered Saline
PN: Pronucleus
PTM: Post-Translational Modification
Q1: First Quartile
Q2: Second Quartile
Q3: Third Quartile
Q4: Fourth Quartile
RIPA: Radioimmunoprecipitation Assay
RNA: Ribonucleic Acid
RT: Room Temperature
SD: Standard Deviation
SPSS: Statistical Package for the Social Sciences
TBS: Tris-Buffered Saline
TBST: Tris-Buffered Saline with Tween 20
t2: Time to 2-cell stage
t3: Time to 3-cell stage
t4: Time to 4-cell stage
t5: Time to 5-cell stage
t6: Time to 6-cell stage
t7: Time to 7-cell stage
t8: Time to 8-cell stage
tPNf: Time to Pronuclear Fading
TFF: Total Fertilization Failure
TPS: Total Protein Stain
VCM: Volume x Concentration x Motility
WHO: World Health Organization
WMO: Wet Medisch wetenschappelijk Onderzoek (Medical research act)
