# Supplementary material for: Sperm H3K9me3 levels are associated with embryo developmental dynamics and biochemical pregnancy in IVF patients with normozoospermia
Source: Reprod Biol Endocrinol. 2025 Dec 3;24:3. doi: 10.1186/s12958-025-01505-w (PMC12781357; doi:10.1186/s12958-025-01505-w)
Supplement: Supplementary file 1 — Supplementary figure legends 1–4 and supplementary tables 1–11. [file 12958_2025_1505_MOESM1_ESM.docx]

**Supplementary material**

**Supplemental figure 1: Representative images of Western blot with 10 patient samples confirming antibody specificity and protein size. A)** Image after total protein staining (TPS), showing a strong and variable band around 65kDa . **B)** Merged image of the blot after incubation with the primary and secondary antibodies and imaging. In red (700nm channel) Histone H3 and the Dual colour protein ladder. In green (800nm channel) H3K9me3. **C)** Single channel image of the 700nm channel showing the result of histone H3 antibody. **D)** Single channel image of the 800nm channel showing the result of H3K9me3 antibody.

**Supplemental figure 2: Variation in histone H3 levels and H3K9me3/H3 ratios between 80/40% and 90/45% sperm processing gradient**. **A-C)** Boxplots histone H3 (80/40% (n = 41) vs. 90/45% (n= 56)), H3K9me3/H3 ratio, Fertilization rate and Embryo utilization rate (80/40% (n = 43) vs. 90/45% (n= 56)) are presented as a median with a 25 and 75 percentile, whiskers are presented as a minimum a maximum. Data was not normally distributed in all tested parameters. **A)** The 80/40% and 90/45% gradient showed significant differences in histone H3 (Mann Whitney U-test, p=0.0002). **B-C)** The H3K9me3/H3 ratio and fertilization rate and showed no significant differences.

**Supplemental figure 3: Spearman rank correlation between histone H3 level, H3K9me3/H3 ratio, male age and VCM.** **A-E)** Each individual dot in the graphs represents a patient. **A-B)** No significant correlations were found between male age and histone H3 (ρ = 0.078, p-value 0.449) or the H3K9me3/H3 ratio (ρ = -0.112, p-value 0.230). **C-D)** Additionally, no significant correlations were observed for VCM and histone H3 (ρ = 0.110, p-value 0.283) or H3K9me3 (ρ = 0.173, p-value 0.087). **E)** A significant positive correlation was observed between histone H3 level and the H3K9me3/H3 ratio (ρ = 0.205, p-value 0.044).

**Supplemental figure 4: Boxplots of embryo morphokinetic annotations up to t8 (time to 8-cells stage) per H3K9me3/H3 quartile**. X-axis represents the time in hours. Each box represents the median and interquartile range (IQR). Whiskers show the minimum and maximum of the data.

**Supplemental table 1: Baseline characteristics of all patient couples with successful histone H3 and H3K9me3/H3 ratio measurements in the sperm sample**

|  | **Baseline characteristics** (n = 99) |
| --- | --- |
| **Male age** | 37.08 [33.00 – 40.16] |
| **VCM** | 119.88 [73.50 – 198.09] |
| **Female age** | 35.83 [32.00 – 39.08] |
| **Main diagnosis** |  |
| Unexplained infertility | 40 (41%) |
| Fallopian tube factor | 20 (20%) |
| Irregular menstrual cycle | 7 (7%) |
| PCOS | 4 (4%) |
| Endometriosis | 20 (20%) |
| Uterine factor | 2 (2%) |
| Other | 6 (6%) |
| **Number of oocytes** | 8.00 [5.00 -11.00] |
| **Fertilization rate** | 66.67 [45.45 – 80.00] |
| **Embryo Utilization Rate** | 73.21 [50.00 – 100.00] |
| **Cultured in Embryoscope** |  |
| Yes | 81 (82%) |
| No | 18 (18%) |
| **Day of fresh embryo transfer** |  |
| No transfer | 9 (9%) |
| Day 3 | 26 (26%) |
| Day 5 | 64 (65%) |
| **Day of cryopreservation** |  |
| Day 4 | 21 (21%) |
| Day 5/6 | 78 (79%) |
| **Embryo’s transferred (Fresh)** |  |
| 0 | 9 (9%) |
| 1 | 81 (82%) |
| 2 | 9 (9%) |
| **Biochemical pregnancy fresh transfer (n=90)** |  |
| Yes | 40 (44%) |
| No | 50 (56%) |
| **Ongoing pregnancy fresh transfer (n=90)** |  |
| Yes | 32 (36%) |
| No | 58 (64%) |
| **Frozen embryo transfer (FET) (n=97)** |  |
| Yes | 52 (54%) |
| No | 45 (46%) |
| **Cumulative biochemical pregnancy (within the same cycle) (n=97)** |  |
| Yes | 55 (57%) |
| No | 42 (43%) |
| **Cumulative ongoing pregnancy (within the same cycle) (n=97)** |  |
| Yes | 40 (41%) |
| No | 57 (59%) |

Data are reported as median [interquartile range (IQR)]. p-value of <0.05 was considered significant. Abbreviations: VCM. Volume*concentration*motility; PCOS, Poly cystic ovary syndrome.

**Supplemental table 2: Baseline characteristics of all initially included patients**

|  | **Baseline characteristics (n = 99)** | **Missing data**  **(n=14)** | **p-value** |
| --- | --- | --- | --- |
| **Male age** | 37.08 [33.00 – 40.16] | 32.38 [29.31 – 40.05] | 0.135 |
| **VCM** | 119.88 [73.50 – 198.09] | 67.74 [56.89 – 124.32] | **0.029** |
| **Female age** | 35.83 [32.00 – 39.08] | 33.00 [31.54 – 38.86] | 0.536 |
| **Main couple diagnosis** |  |  |  |
| Unexplained infertility | 40 | 5 | 0.984 |
| Fallopian tube factor | 20 | 4 |  |
| Irregular menstrual cycle | 7 | 1 |  |
| PCOS | 4 | 0 |  |
| Endometriosis | 20 | 3 |  |
| Uterine factor | 2 | 0 |  |
| Other | 6 | 1 |  |
| **Number of oocytes** | 8.00 [5.00 -11.00] | 7.50 [6.00 – 10.00] | 0.916 |
| **Fertilization rate** | 66.67 [45.45 – 80.00] | 78.89 [64.29 – 83.93] | 0.080 |
| **Embryo Utilization Rate** | 73.21 [50.00 – 100.00] | 50.00 [39.38 – 100.00] | 0.223 |
| **Cultured in Embryoscope** |  |  |  |
| Yes | 81 (82%) | 13 | 0.458 |
| No | 18 (18%) | 1 |  |
| **Day of fresh embryo transfer** |  |  |  |
| No transfer | 9 | 1 | 0.525 |
| Day 3 | 26 | 5 |  |
| Day 5 | 64 | 8 |  |
| **Day of cryopreservation** |  |  |  |
| Day 4 | 21 | 3 | 1.000 |
| Day 5/6 | 78 | 11 |  |
| **Embryo’s transferred (Fresh)** |  |  |  |
| 0 | 9 | 1 | 0.103 |
| 1 | 81 | 9 |  |
| 2 | 9 | 4 |  |
| **Biochemical pregnancy fresh transfer** |  |  |  |
| Yes | 40 (44%) | 7 | 0.564 |
| No | 50 (56%) | 6 |  |
| **Ongoing pregnancy fresh transfer** |  |  |  |
| Yes | 32 (36%) | 5 | 1.000 |
| No | 58 (64%) | 8 |  |
| **Cumulative biochemical pregnancy (within the same cycle)** |  |  |  |
| Yes | 55 (57%) | 8 | 1.000 |
| No | 42 (43%) | 5 |  |
| **Cumulative ongoing pregnancy (within the same cycle)** |  |  |  |
| Yes | 40 (41%) | 7 | 0.552 |
| No | 57 (59%) | 6 |  |

Data are reported as median [interquartile range (IQR)]. p-value of <0.05 was considered significant. Abbreviations: VCM. Volume*concentration*motility; PCOS, Poly cystic ovary syndrome.

**Supplemental table 3: Nucleolar precursor body clustering dynamics analysis at the pronuclear stage per H3K9me3/H3 quartile.**

| Nucleolar precursor body clustering dynamics  (n=428 zygotes) | **H3K9me3/H3 ratio Q1**  **(0.014 – 0.075)** | **H3K9me3/H3 ratio Q2**  **(0.075 – 0.133)** | **H3K9me3/H3 ratio Q3**  **(0.133 – 0.260)** | **H3K9me3/H3 ratio Q4**  **(0.260 – 2.498)** | **p-value** |
| --- | --- | --- | --- | --- | --- |
| Class 1 | 20 (27%) | 35 (28%) | 48 (42%) | 28 (24%) | **0.001** |
| Class 2 | 15 (21%) | 43 (34%) | 27 (24%) | 28 (24%) |  |
| Class 3 | 17 (23%) | 23 (18%) | 29 (26%) | 28 (24%) |  |
| Class 4 | 21 (29%) | 25 (20%) | 9 (8%) | 32 (28%) |  |

A p-value of <0.05 was considered significant, in bold.

**Supplemental table 4: Nucleolar precursor body clustering analysis at the pronuclear stage per H3K9me3/H3 quartile.**

| Nucleoli clustering  (n=432 zygotes) | **H3K9me3/H3 ratio Q1**  **(0.014 – 0.075)** | **H3K9me3/H3 ratio Q2**  **(0.075 – 0.133)** | **H3K9me3/H3 ratio Q3**  **(0.133 – 0.260)** | **H3K9me3/H3 ratio Q4**  **(0.260 – 2.498)** | **p-value** |
| --- | --- | --- | --- | --- | --- |
| Clustered | 35 (48%) | 76 (60%) | 75 (66%) | 56 (47%) | **0.011** |
| Unclustered | 38 (52%) | 50 (40%) | 39 (34%) | 63 (53%) |  |

A p-value of <0.05 was considered significant, in bold.

**Supplemental table 5: Cleavage pattern analysis of zygotes per H3K9me3/H3 quartile.**

| **Cleavage pattern analysis** (n=439 zygotes) | **H3K9me3/H3 ratio Q1**  **(0.014 – 0.075)** | **H3K9me3/H3 ratio Q2**  **(0.075 – 0.133)** | **H3K9me3/H3 ratio Q3**  **(0.133 – 0.260)** | **H3K9me3/H3 ratio Q4**  **(0.260 – 2.498)** | **p-value** |
| --- | --- | --- | --- | --- | --- |
| **Direct unequal cleavage** | 20 (26%) | 28 (22%) | 27 (23%) | 30 (25%) | 0.903 |
| **Normal cleavage division** | 57 (74%) | 99 (78%) | 89 (77%) | 89 (75%) |  |

Embryos were defined as direct unequal cleaving (DUC) if they needed 5 h or less during the cleavage interval t3-t2. A p-value of <0.05 was considered significant, in bold.

**Supplemental table 6: Logistic regression analysis with H3K9me3/H3 ratio as continuous variable and main determinant and cumulative biochemical pregnancy as outcome.**

|  |  |  |  | **95% C.I. for OR** | |
| --- | --- | --- | --- | --- | --- |
|  | **Beta** | **OR** | **p-value** | **lower** | **Upper** |
| H3K9me3/H3 ratio | 0.420 | 1.522 | 0.462 | 0.497 | 4.660 |
| Female age | -0.083 | 0.920 | 0.079 | 0.838 | 1.010 |

A p-value of <0.05 was considered significant, in bold. The addition of female age as potential confounder. Abbreviations: OR, odds ratio; C.I., confidence interval.

**Supplemental table 7: Logistic regression analysis with histone H3 as continuous variable and main determinant and cumulative biochemical pregnancy as outcome.**

|  |  |  |  | **95% C.I. for OR** | |
| --- | --- | --- | --- | --- | --- |
|  | **Beta** | **OR** | **p-value** | **lower** | **Upper** |
| Histone H3 | 0.149 | 1.161 | 0.454 | 0.786 | 1.715 |
| Female age | -0.078 | 0.925 | 0.099 | 0.843 | 1.015 |

A p-value of <0.05 was considered significant, in bold. The addition of female age as potential confounder.
Abbreviations: OR, odds ratio; C.I., confidence interval.

**Supplemental table 8:** **Baseline characteristics of patients IVF-cycles resulting in cumulative ongoing pregnancy or no pregnancy.**

|  | **No ongoing pregnancy** (n = 57) | **Cumulative ongoing pregnancy** (n = 40) | **p-value** |
| --- | --- | --- | --- |
| **Male age** | 37.08 [33.96 – 40.21] | 37.67 [33.96 – 40.70] | 0.461 |
| **Female age** | 37.08 [32.96 – 39.71] | 34.38 [31.00 – 37.95] | 0.097 |
| **Main couple diagnosis** |  |  |  |
| Unexplained infertility | 20 | 18 | 0.302 |
| Fallopian tube factor | 10 | 10 |  |
| Irregular menstrual cycle | 5 | 2 |  |
| PCOS | 1 | 3 |  |
| Endometriosis | 15 | 5 |  |
| Uterine factor | 1 | 1 |  |
| Other | 5 | 1 |  |
| **Number of oocytes** | 6.00 [5.00 – 10.00] | 9.50 [5.00 – 12.75] | 0.074 |
| **Fertilization rate** | 62.50 [40.00 – 77.50] | 76.70 [54.06 – 80.00] | **0.039** |
| **Embryo Utilization Rate** | 66.67 [50.50 – 96.43] | 76.39 [66.67 – 100.00] | 0.088 |
| **Embryo transfer policy** |  |  |  |
| SET | 51 | 37 | 0.307 |
| DET | 7 | 2 |  |
| **Histone H3** | 0.015 [0.010 – 0.020] | 0.013 [0.010 – 0.021] | 0.868 |
| **H3K9me3/H3 ratio** | 0.13 [0.07 – 0.26] | 0.13 [0.09 – 0.29] | 0.907 |
| **Day of cryopreservation** |  |  |  |
| Day 4 | 17 | 9 | 0.484 |
| Day 5,6 | 36 | 28 |  |

Data are reported as median [interquartile range (IQR)]. Abbreviations: PCOS, Poly cystic ovary syndrome; SET, Single embryo transfer; DET, Double embryo transfer.

**Supplemental table 9: Logistic regression analysis with H3K9me3/H3 ratio as continuous variable and main determinant and cumulative ongoing pregnancy as outcome.**

|  |  |  |  | **95% C.I. for OR** | |
| --- | --- | --- | --- | --- | --- |
|  | **Beta** | **OR** | **p-value** | **lower** | **Upper** |
| H3K9me3/H3 ratio | -0.338 | 0.713 | 0.590 | 0.209 | 2.437 |
| Female age | -0.063 | 0.939 | 0.176 | 0.858 | 1.028 |

A p-value of <0.05 was considered significant, in bold. The addition of female age as potential confounder.
Abbreviations: OR, odds ratio; C.I., confidence interval.

**Supplemental table 10: Logistic regression analysis with histone H3 as continuous variable and main determinant and cumulative ongoing pregnancy as outcome.**

|  |  |  |  | **95% C.I. for OR** | |
| --- | --- | --- | --- | --- | --- |
|  | **Beta** | **OR** | **p-value** | **lower** | **Upper** |
| **Histone H3** | 0.086 | 1.090 | 0.634 | 0.764 | 1.557 |
| **Female age** | -0.062 | 0.940 | 0.179 | 0.859 | 1.029 |

A p-value of <0.05 was considered significant, in bold. The addition of female age as potential confounder.
Abbreviations: OR, odds ratio; C.I., confidence interval.

**Supplemental table 11: Logistic regression analysis with H3K9me3/H3 ratio quartiles as main determinant and cumulative ongoing pregnancy as outcome.**

|  |  |  |  | **95% C.I. for OR** | |
| --- | --- | --- | --- | --- | --- |
|  | **Beta** | **OR** | **p-value** | **lower** | **Upper** |
| **H3K9me3/H3 ratio quartiles** |  |  |  |  |  |
| Quartile 1 vs. Quartile 3 | -0.198 | 0.82 | 0.742 | 0.25 | 2.68 |
| Quartile 2 vs. Quartile 3 | 0.545 | 1.73 | 0.354 | 0.54 | 5.47 |
| Quartile 4 vs. Quartile 3 | 0.277 | 1.32 | 0.636 | 0.42 | 4.16 |
| **Female age** | -0.062 | 0.94 | 0.187 | 0.86 | 1.03 |

A p-value of <0.05 was considered significant, in bold. Odds ratios adjusted for female age.
Abbreviations: OR, odds ratio; C.I., confidence interval. Q3 was selected as reference category based on exploratory findings. Results should be interpreted with caution due to small group sizes.
