# Supplementary figures and images for: Sperm H3K9me3 levels are associated with embryo developmental dynamics and biochemical pregnancy in IVF patients with normozoospermia
Source: Reprod Biol Endocrinol. 2025 Dec 3;24:3. doi: 10.1186/s12958-025-01505-w (PMC12781357; doi:10.1186/s12958-025-01505-w)

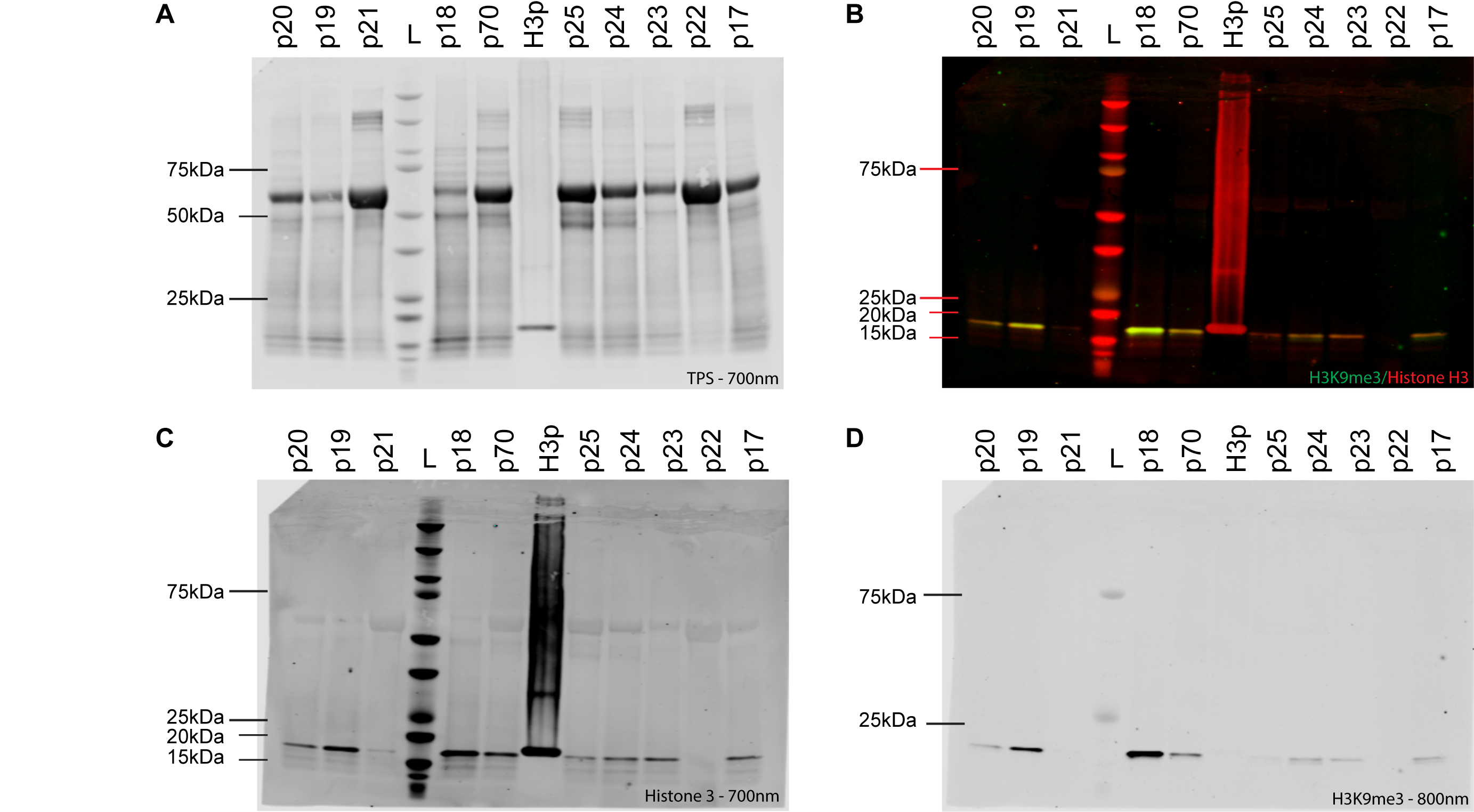

Supplement: Supplementary file 2 — Figure S1. Representative images of Western blot with 10 patient samples confirming antibody specificity and protein size. [file 12958_2025_1505_MOESM2_ESM.tif]

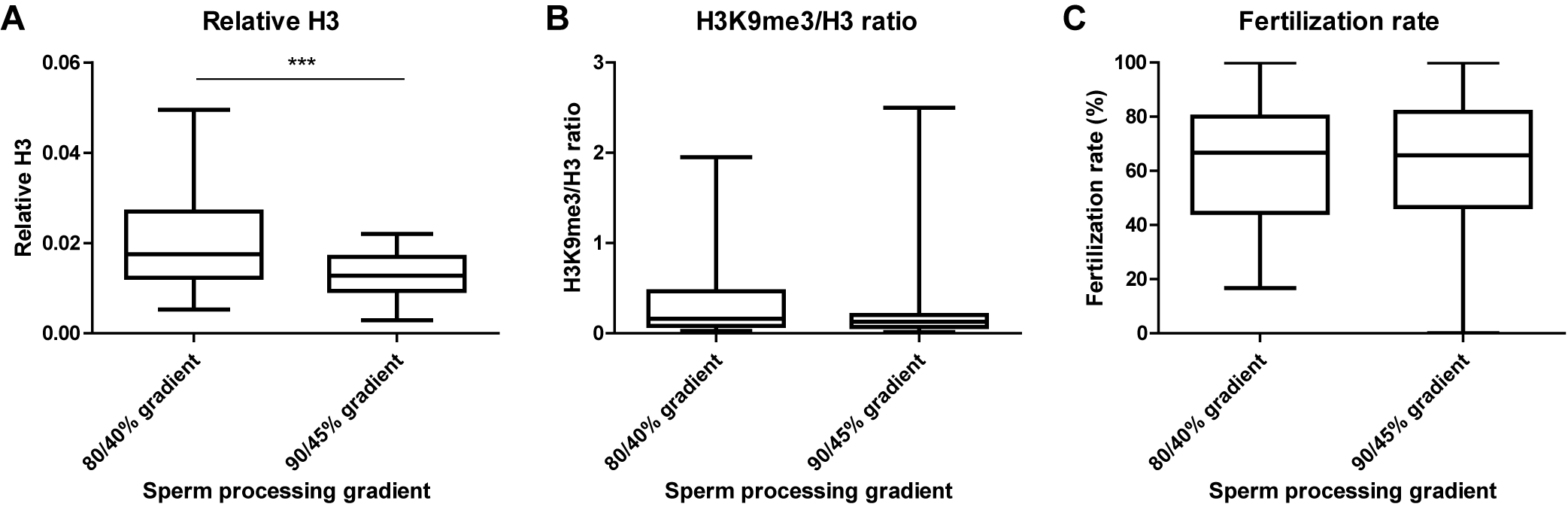

Supplement: Supplementary file 3 — Figure S2. Variation in histone H3 levels and H3K9me3/H3 ratios between 80/40% and 90/45% sperm processing gradient. [file 12958_2025_1505_MOESM3_ESM.tif]

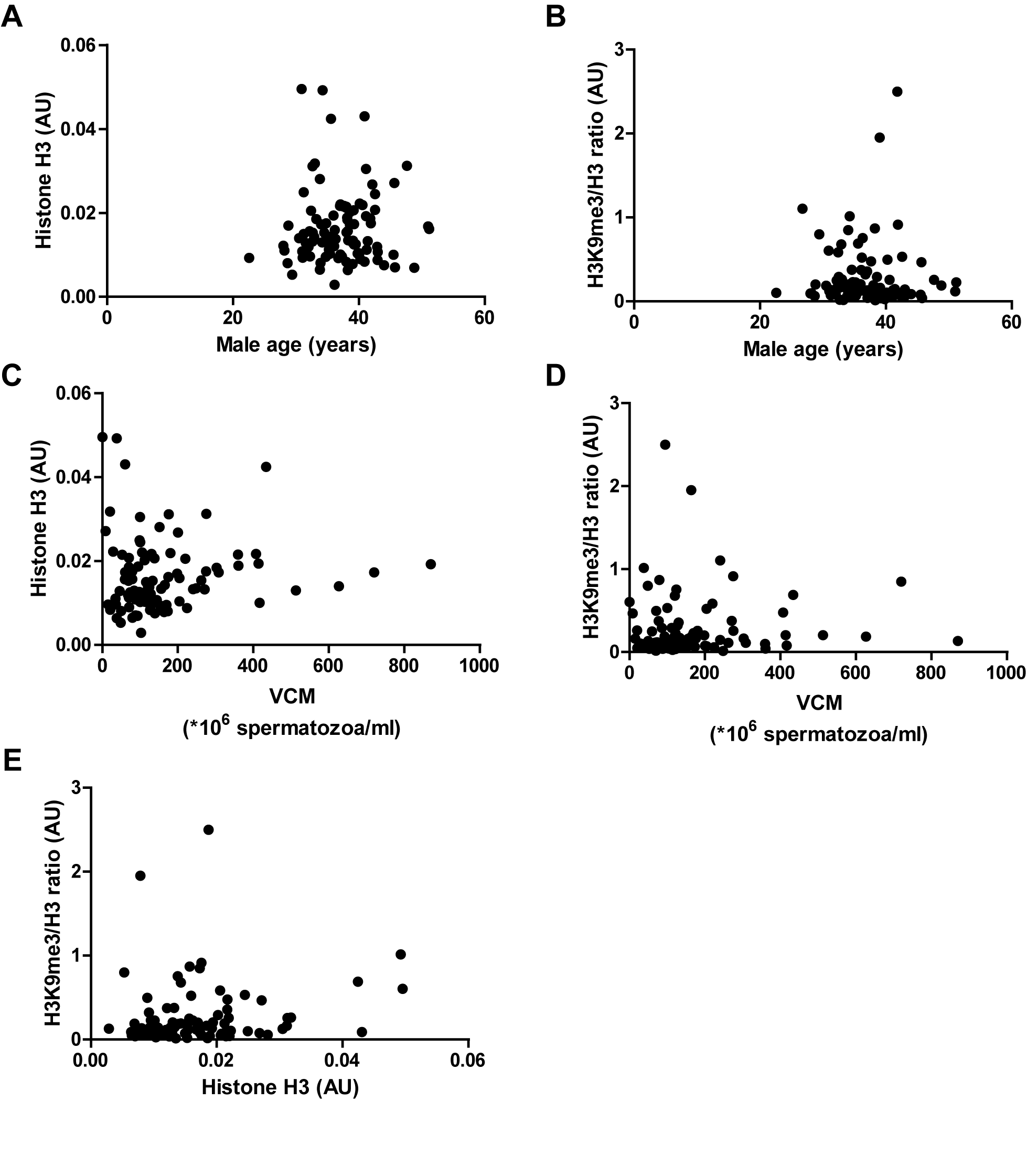

Supplement: Supplementary file 4 — Figure S3. Spearman rank correlation between histone H3 level, H3K9me3/H3 ratio, male age and VCM. [file 12958_2025_1505_MOESM4_ESM.tif]

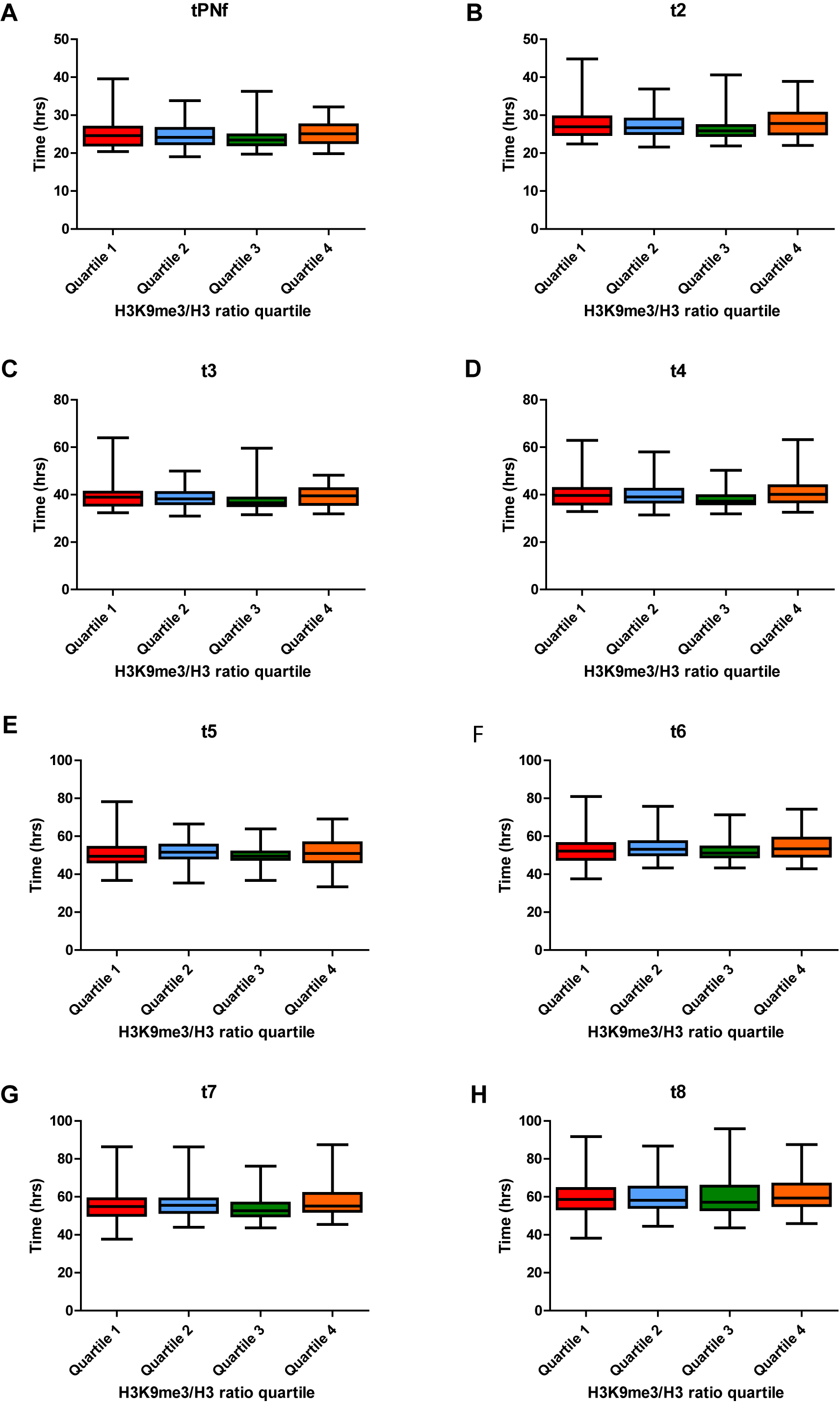

Supplement: Supplementary file 5 — Figure S4. Boxplots of embryo morphokinetic annotations up to t8 (time to 8-cells stage) per H3K9me3/H3 quartile. [file 12958_2025_1505_MOESM5_ESM.tif]
